# Cumulative secondary systemic metabolic cerebral insults are associated with disease severity and mortality in neonatal hypoxic–ischaemic encephalopathy: a retrospective study

**DOI:** 10.3389/fped.2026.1781388

**Published:** 2026-04-30

**Authors:** Hélèna Garnaud, Yohan Soreze, Eléonore Blondiaux, Gilles Kayem, Clément Chollat, Pierre-Louis Leger, Jerome Rambaud, Julia Guilbert, Isabelle Guellec

**Affiliations:** 1 Neonatal Intensive Care Medicine, Port-Royal University Hospital, Assistance Publique des Hôpitaux de Paris, Paris, France; 2 Neonatal and Pediatric Intensive Care Unit, Trousseau University Hospital, Assistance Publique des Hôpitaux de Paris, Paris, France; 3 Pediatric Radiology Unit, Trousseau University Hospital, Assistance Publique des Hôpitaux de Paris, Paris, France; 4 Obstetric Unit, Trousseau University Hospital, Assistance Publique des Hôpitaux de Paris, Paris, France; 5 Neonatal Intensive Care Medicine, University Hospital Nice Cote D’Azur, Nice, France; 6 Department of Medicine, Nice Côte D’Azur university, Nice, France

**Keywords:** brain lesions, hypoxic-ischemic perinatal event, neonatal encephalopathy, neonatal mortality, secondary cerebral insult

## Abstract

**Background:**

Secondary systemic cerebral metabolic insults (mSSCI), including abnormalities of glucose, sodium, and carbon dioxide, may exacerbate brain injury in neonatal hypoxic–ischaemic encephalopathy (HIE). Their burden and prognostic value remain poorly described. We aimed to assess the frequency of mSSCI and their association with disease severity and mortality.

**Methods:**

This single-centre observational study included all newborns admitted for HIE between 2018 and 2020. mSSCI were defined as at least one abnormal blood glucose, sodium, or carbon dioxide value measured from routine samples during the first three days of life. A cumulative mSSCI score was calculated as the sum of the six possible abnormalities. Associations between mSSCI burden, markers of perinatal asphyxia, resuscitation characteristics, and mortality were explored using univariate analyses.

**Results:**

Among the 109 newborns, 106 received therapeutic hypothermia within 6 h of birth; 12 developed neonatal seizures, 32 had brain lesions on MRI, and 10 died. 22% experienced no mSSCI, 35% had one, 28% had two, and 15% had three or more. Increasing cumulative mSSCI was significantly associated (*p* < 0.01) with greater HIE severity (8.3% of newborns without mSSCI to 53.8% with ≥3 mSSCI were Sarnat III stage), higher lactate levels at birth and lower 5-minute Apgar scores (*p* < 0.01). The cumulative number of mSSCI was significantly associated with the severity of individual metabolic disturbances. Mortality increased significantly with cumulative mSSCI, from 0/24 in newborns without mSSCI to 5/16 (31.3%) in those with ≥3 mSSCI.

**Conclusions:**

Most newborns with HIE experienced metabolic systemic insults. Accumulation of mSSCI was associated with encephalopathy severity and mortality. Further studies are needed to determine whether mSSCI are markers of illness severity or modifiable targets for neuroprotective strategies.

## Background

Hypoxic-ischemic encephalopathy (HIE) in newborns is an important cause of death and neurodevelopmental sequelae despite intensive care and therapeutic hypothermia (TH) (1). Secondary systemic cerebral insults are known to be associated with increased cerebral lesions in patients with cerebral trauma (2) or with vascular accidents in adults (3) and children (4). The challenge for neonatologists is to minimize these potential aggressions to improve neonatal outcomes.

Few metabolic secondary systemic cerebral insults (mSSCI) have been studied individually in neonates with HIE. Both neonatal hyperglycemia and hypoglycemia are associated with an increased risk of death and/or severe neurological disability (5–7). Studies have shown an association between hypocapnia and impaired neurodevelopment at 2 years of age (8–10). Hyponatremia is more common in patients with HIE, and the more severe the HIE, the more severe the hyponatremia (11, 12). Positive fluid balance during TH is associated with death and moderate-to-severe brain injuries on magnetic resonance imaging (13). Studies have looked at mSSCI individually, but no study has investigated the combination of mSSCI. Moreover, little is known about their prevalence or their association with short-term neurological outcome.

We aimed to describe the frequency of mSSCI in neonates with HIE during the first three days of life and to assess their association with resuscitation care and mortality in newborns with neonatal encephalopathy.

## Methods

### Population

This study was a single-center retrospective observational study of newborns hospitalized on the first day of life for HIE in the neonatal intensive care unit (NICU) of Trousseau Hospital (Assistance Publique, Hôpitaux de Paris) in France during the three years period of 2018–2020. The data were collected by reviewing the medical records of all patients with HIE (identified by querying our electronic database).

The diagnostic criteria for moderate or severe HIE were those published by the French Society of Neonatology (14), i.e., early neurological distress with clinical signs of moderate or severe neurologic encephalopathy on a standardized neurological examination performed by a senior investigator in the first hours of life in a context of perinatal asphyxia including laboratory signs of severe asphyxia or of moderate or unknown levels of asphyxia with adverse perinatal events.

### Maternal and neonatal data collection

We collected maternal data such as the delivery method (cesarean, instrumental, or vaginal), presence of an NICU at the maternity hospital of birth (yes/no), and the presence of maternal or gestational diabetes. The neonatal data collected included gestational age at birth, sex, birthweight, cord pH and lactate levels, and Apgar scores at one and five minutes. We collected the neurological evaluation with the Sarnat score (15) at admission to Trousseau Hospital.

Information about intensive care treatment during the first three days of life, corresponding to the period of neuroprotection by TH, included glucose infusion (gram per kilograms per days), salt intake (mEq/kg/day), and infusion volume (water intake mL/kg/day) for both enteral and parenteral treatment. We also collected invasive ventilation parameters at day 1 (D1) of life, vasopressor use (i.e., epinephrine, dobutamine, or norepinephrine regardless of dose), and insulin treatment.Urea (mmol/L), creatininemia (µmol/L), and diuresis (mL per kilograms per days) were collected during routine care at 12 h of life (H12), H24, H48, and H72.

### Metabolic secondary cerebral insults

mSSCI were defined as abnormalities concerning measurements of glycemia, natremia, and/or capnia during routine sampling at least once during the first three days of life. The unit protocol calls for a blood sample at admission, another sample between 12 and 24 h of life, one at H48 and one at H72. Glycemia was measured by capillary blood, capnia by capillary or venous blood gas and natremia by venous blood ionogram. We defined hypoglycemia as a blood glucose level below 2 mmol/L, hyperglycemia as a blood glucose level above 10 mmol/L; hyponatremia as serum sodium below 130 mmol/L, hypernatremia as serum sodium above 150 mmol/L; and hypocapnia as partial pressure of carbon dioxide (pCO2) in venous or capillary blood below 30 mmHg, and hypercapnia as pCO2 in venous or capillary blood above 70 mmHg (values for capnia were corrected according to the newborn's temperature).

We defined the total number of types of mSSCI as the sum of the types of mSSCI present (among hypoglycemia, hyperglycemia, hyponatremia, hypernatremia, hypocapnia, and hypercapnia) whatever the repetition of the same type of mSSCI was (for example, an infant with one measurement defined as hypoglycemia and two measurements of hyperglycemia has a total of two types of mSSCI — hypoglycemia and hyperglycemia).

#### Primary outcome

Our primary outcome was the frequency of mSSCI in our population.

#### Secondary outcome

Our secondy outcomes were defined as mortality and depth of the mSSCI. Mortality was defined as death before hospital discharge.

The depth of the mSSCI was defined as the lowest and the highest level of blood glucose, capnia and natremia taken during the first three days of management.

### Statistical analysis

Results were presented as counts and percentages or medians and their associated standard deviations. We used Fisher's exact test and conducted univariate analysis to assess outcome among newborns with and without mSSCI and among the total number of patients with mSSCI. The ANOVA (Mann Whitney) test was used to compare continuous variables. Results were considered significant with a *p*-value <0.05 with a power of 80%. The statistical analysis was performed with Stata SE 13 software (College Station, TX, USA).

We used the STROBE reporting guideline to draft this manuscript, and the STROBE reporting checklist when editing, included in supplement.

## Results

**Figure 1:**
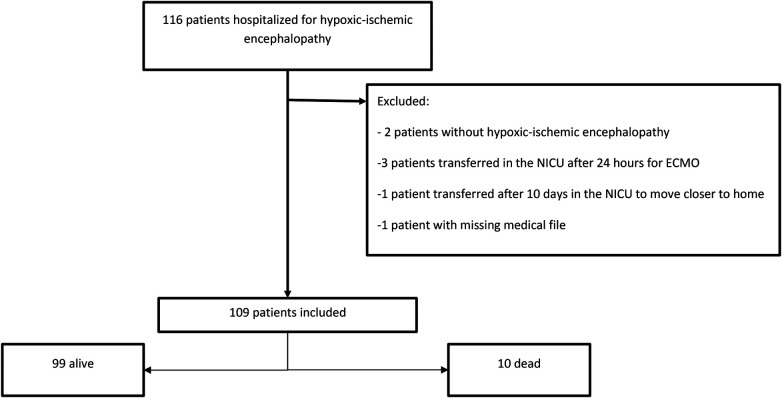
Flow chart.

Among the 116 newborns hospitalized in the NICU during the study period, 109 were included, 1 had a medical record with missing data, 2 were excluded because they did not have HIE, and 4 because they were not admitted to our NICU within the first 24 h of life. The primary outcome was known for 106 patients (Figure 1). mSSCI data were missing for three patients: two had two missing data items (one related to glycemia and the other to capnia), and one patient one missing data item (hypoglycemia).

**Figure 2:**
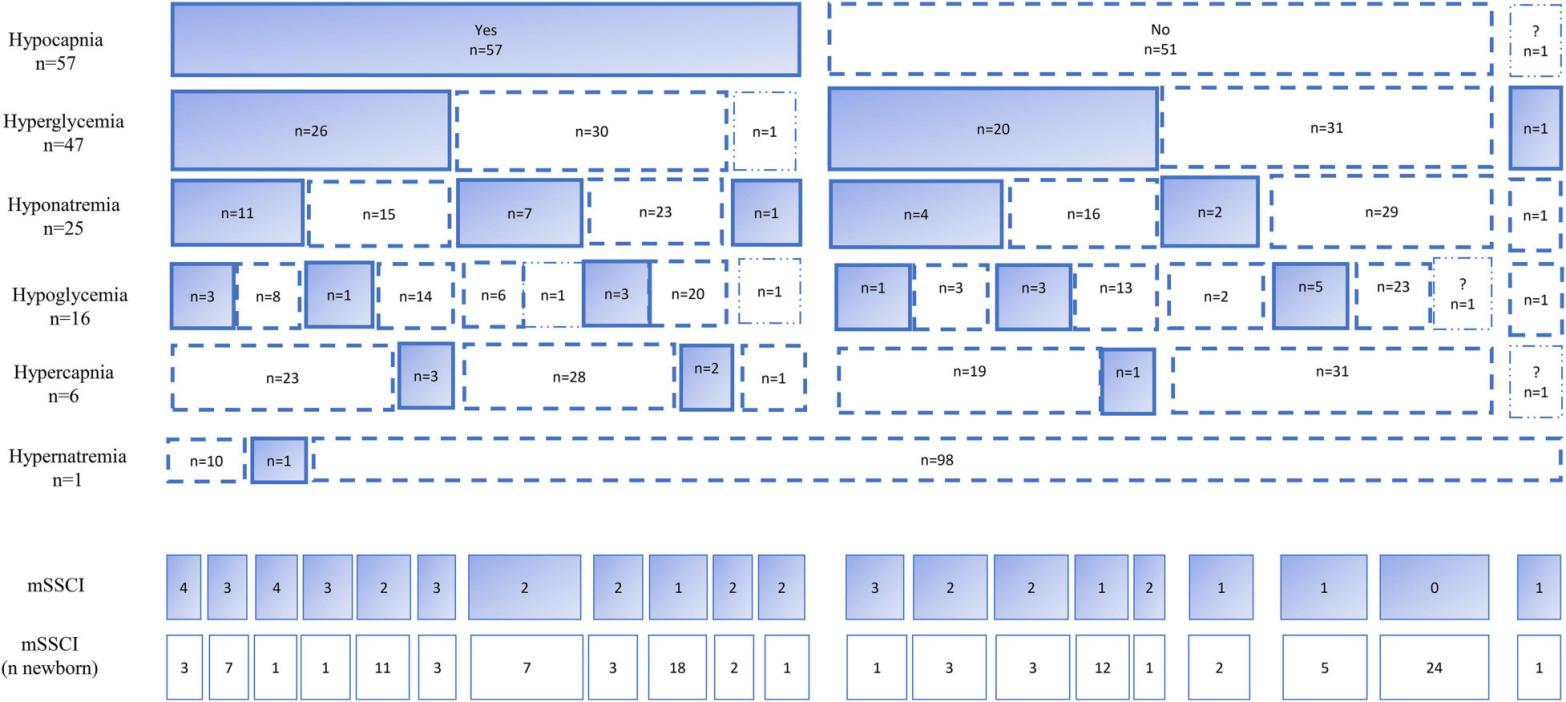
Distribution of mSSCI.

Among the 109 newborns included, 106 received therapeutic hypothermia within 6 h of birth; 12 developed neonatal seizures, 32 had brain lesions on MRI, and 10 died. Hypocapnia was found in 57 patients (52%), hyperglycemia in 47 (43%), hyponatremia in 25 (23%), hypoglycemia in 16 (15%), hypercapnia in 6 (6%), and hypernatremia in one (1%). Cumulative mSSCI was 0 mSSCI in 24 newborns (22%), 1 mSSCI in 38 (35%), 2 mSSCI in 31 (28%), 3 mSSCI in 12 (11%) and 4 mSSCI in 4 patients (4%) (Figure 2).

We found no statistical association between obstetric variables and the cumulative number of mSSCI. Apgar scores at M1 and M5 were significantly (*p* < 0.01) lower as the number of mSSCI types rose. HIE severity was significantly associated with higher numbers of mSSCI: Sarnat III was the grade for 8.3% of those with no mSSCI, 24.3% for those with one mSSCI, 42.3% for those with 2 mSSCI, and 53.8% for those with ≥3 mSSCI. The need for vasopressors increased and the mean maximum serum lactate value were significantly higher as the number of mSSCI types rose: 12.2 mmol/L (± 4.1) in the group with no mSSCI, 11.6 mmol/L (± 4.6) with one, 14.0 mmol/L (±4.5) with 2, and 17.0 mmol/L (± 4.4) with 3 or more (*p* < 0.01).

**Figure 3:**
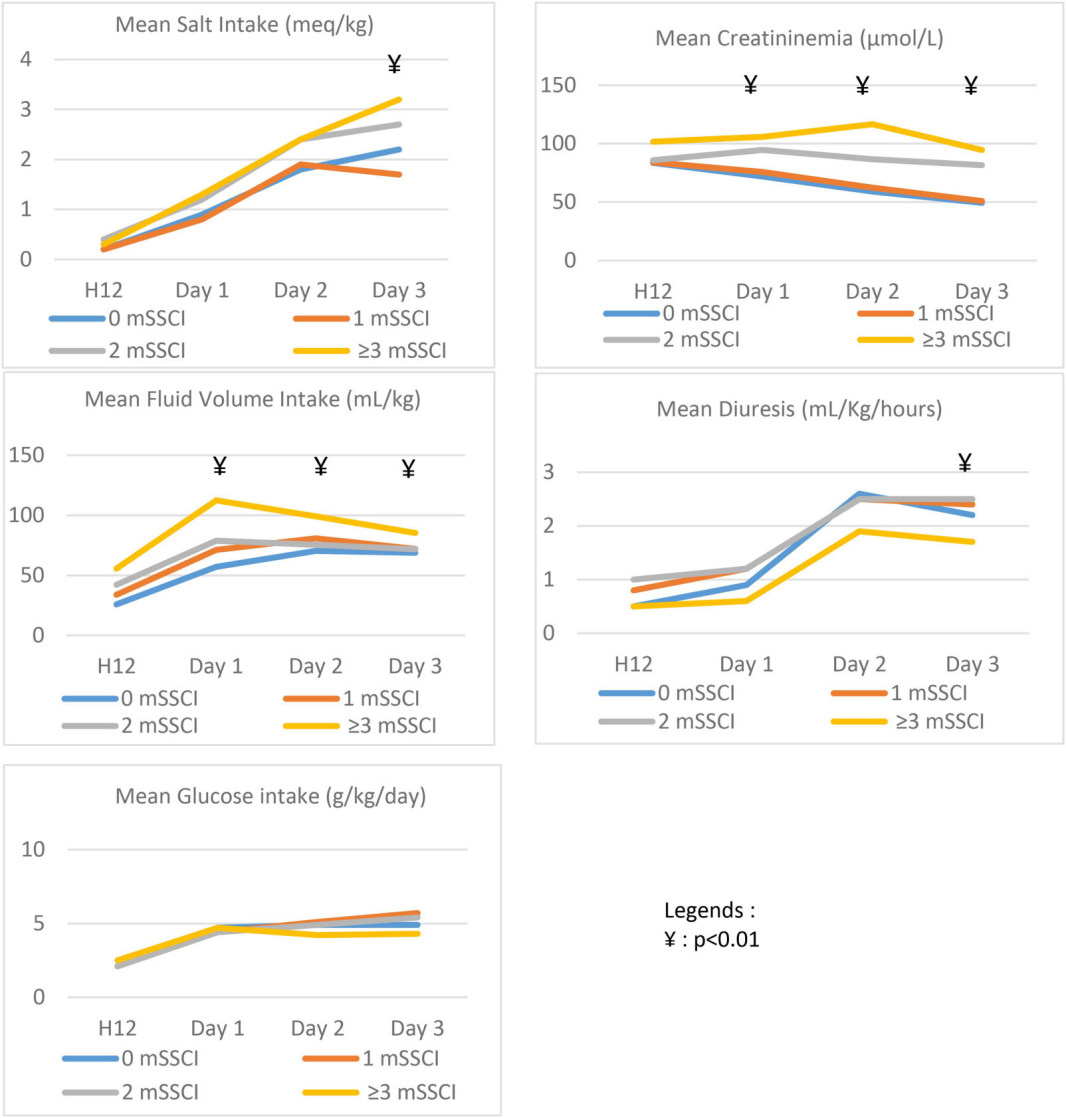
Contributing variables for mSSCI interpretation by days.

Concerning the depth of metabolic disorders (Table 1), this was significantly associated with the cumulative number of mSSCI. The maximum glycemic value was significantly higher with the highest number of mSSCI types [6.6 (±1.4) mmol/L at no mSSCI to 17.9 (± 8.9 mmol/L) at three or more]. The minimum value of natremia was also significantly lower in the groups with multiple mSSCI: 133.8 mmol/L (± 2.3) for the group with no mSSCI, 133.2 mmol/L (± 2.9) for the group with one, 131.7 mmol/L (± 4.3) for the group with two, and 128.6 mmol/L (± 4.8) for the group with three or more. For CO2 levels, the minimum value of pCO2 was significantly (*p* < 0.01) lower in patients with more types of mSSCI: from 35.3 mmHg (± 4.5) for no mSSCI to 29.4 mmHg (± 6.6) for one, 25.1 mmHg (± 6.2) for two, and 22.9 mmHg (± 5.3) for three or more. (Table 1). Figure 3 details the contributing variables for mSSCI interpretation by days. Mean glucose intake did not differ significantly among the number of mSSCI at any time point. Water intake was significantly higher in the group with the most mSSCI from H12 to D2. Diuresis did not differ significantly among the mSSCI groups until D3, when it was lowest for the group with three or more mSSCI. In addition, creatininemia were significantly higher from D1 to D3 in patients with more mSSCI.

**Table 1:** Neurological and biological inclusion criteria for moderate or severe hypoxic-ischemic encephalopathy.

Neurological signs	
Moderate	Lethargy, hyper-reflexia, myosis, bradycardia, seizures, hypotonia with weak suck and poor Moro reflex
Severe	Stupor, flaccidity, small to mid-position pupils that react poorly to light, decreased stretch reflexes, hypothermia, or absent Moro reflex
Biological criteria^a^	
Severe biological signs	pH ≤ 7.0 or less or a base deficit ≥ 16 mmol/L or lactate ≥ 11 mmol/L
Moderate/absent biological signs with additional perinatal events	7.0 < pH < 7.15, or 10 ≤ base deficit < 16 mmol/L, or 8 ≤ lactate < 11 mmol/L, or blood gas measurement unavailable with
	- An acute perinatal event (e.g., late or variable decelerations, cord prolapse, cord rupture, uterine rupture, maternal trauma, hemorrhage, or cardiorespiratory arrest - Or an abrupt change in fetal heart rate (FHR), defined as a persistent abnormal FHR after a period of normal tracing: bradycardia or prolonged deceleration, persistent variable decelerations, persistent late decelerations, and reduced heart variability - Or either a 10-min Apgar score of 5 or less or assisted ventilation initiated at birth and continued for at least 10 min.

a Biological criteria indicating asphyxia were provided by umbilical cord blood or any other blood sampled during the first hour after birth.

Concerning neonatal outcome, the mortality rate was significantly higher (*p* < 0.01) in neonates with more mSSCI types: none for those with no mSSCI, 2 (5.3%) for one, 3 (9.7%) for two, and 5 (31.3%) for those with three or more.

## Discussion

Only 22% of our cohort of newborns with HIE in this retrospective study were not exposed to metabolic secondary systemic cerebral insults during their first three days of life. The more severe the HIE, the more mSSCI types they experienced and the more profound their metabolic disorders were. We found a significant association between accumulation of mSSCI types and mortality.

This was a single-center retrospective study. These methodological aspects may be associated with biases. Our single-center study may not be representative of the general population or the general care of newborns with HIE. Nonetheless, this study design, involving consistent care over time and among patients, suggests that the associations were related to the newborns and mSSCI types rather than to center practices. Moreover, very few data were missing, and all were retrieved from the same source — the medical files; this should have limited the information bias. Although the size of our population was large, it was nonetheless too small to perform multivariate analysis.

Our results showed a strong association between mortality and mSSCI, i.e., hyperglycemia, hyponatremia, and hypocapnia. A large Canadian study showed that, in addition to initial condition, hemodynamic failure and renal insufficiency were also associated with the outcome of newborns with HIE (16). These three metabolic pathways were chosen because each has been widely individually explored in the literature (6, 9, 11), is potentially modifiable, and has never been examined from an overall viewpoint. We have chosen to focus only on severe forms of mSSCI that are regularly monitored and are potentially modifiable by adapting care.

The most common definitions for them are: hypoglycemia below 2.6 mmol/L (17, 18), hyperglycemia above 8.3 mmol/L (5, 18), hyponatremia below 135 mmol/L (19), hypernatremia above 145 mmol/L (11), hypocapnia below 40 mmHg in venous blood (20), and hypercapnia above 50 mmHg (20, 21). Our stringent choice of profound threshold should have decreased the frequency of mSSCI events in our population by allowing us to exclude its mild forms. This choice underlines the importance of our finding that only 22% of our population had no mSSCI early after their resuscitation. Prevention of secondary brain damage of systemic origin is well known to improve outcomes after both traumatic brain injury and stroke (2, 4). In population-based studies, the frequency of individual mSSCIs reported is also important: for example, 2/3 of patients in Lytonepal had a glycemic regulation disorder (5).

Both neonatal hyperglycemia and hypoglycemia in patients with HIE are associated with a higher risk of death and/or severe neurological disability (5, 6, 21). Hypoglycemia can lead to convulsions in the short term and neurological sequelae in the long term for all children in the perinatal period (22). The glucose supplementation of newborn in case of hypoglycemia is the subject of a consensus despite questions about threshold of hypoglycemia (17). Hyperglycemia and abrupt variations in blood glucose levels increase adverse outcome in NE (5, 6) In our study, hyperglycemia was treated with insulin in only 25.5% of cases, and sugar intake did not differ significantly between mSSCI groups or over time despite the knowledge of this hyperglycemia. It is possible that hyperglycemia was treated less frequently because of either concern about the lack of knowledge of its long-term consequences or fear of too rapid a decrease when insulin introduction that risks hypoglycemia (23).

Hypocapnia, which affected half of our newborns, was the most common mSSCI in our study. It is known to be associated with adverse outcomes among newborns with HIE (24). Only 70% of our cohort received mechanical ventilation especially among our patients with hypocapnia, 87.5% had mechanical ventilation. Lopez et al. showed that there was more hypocapnia in HIE patients with invasive ventilation (25).

About 25% of cases in our study had hyponatremia. This mSSCI is reported to appear early during the first hours of life and to be associated with the severity of birth asphyxia (11, 26). In our study, it was probably associated with renal insufficiency and with fluid overload. This may be related to haemodynamic instability requiring volume expansion and vasoactive support during management, together with secondary intrinsic renal failure We note that water intake was not well adapted to diuresis in our study and was greater among patients with more mSSCI types. This may be related to haemodynamic instability requiring volume expansion and vasoactive support during management. It may contribute to blood dilution during the first 48 h of life. There may also be an element of acute renal failure, with the increased creatinine during the first 72 h in our study, as previously described by Gupta (27).

Another issue is that as the number of mSSCI types increased, the intensity of each (glycemia, natremia, and capnia) increased although they belong to different metabolic pathways. This raises the question of how to monitor them to improve the prognosis and the control of these metabolic insults. It is not clear from our study whether the total number of mSSCI types was a risk factor or a confounding factor related to the outcome.

Indeed, one of the questions raised in studies concerning the associations between mSSCI and mortality is whether mSSCI serves as a marker of severity or whether they directly contribute to worsening prognosis in patients. We didn't found any obstetrical context difference in our study but our most severely ill newborns exhibited the highest number of mSSCI. The limited sample size does not allow us to draw conclusions regarding the association—rather than any causal relationship—between the presence of mSSCI and outcomes. In the context of adult critical care, it has been shown that limiting mSSCI was associated with an improvement in prognosis. To date, this study is the first in neonatology to quantify various mSSCI and demonstrate their association with the risk of death. Alerting neonatologists to the management of these factors may potentially improve the prognosis of these brain-injured patients. In 2021, Wintermark proposed a care practice bundle to optimize the global management of patients with NE, based on a review of the literature (28).

## Conclusions

Metabolic secondary systemic cerebral insults affected 80% of neonates hospitalized for NE. The cumulative number of mSSCI types was significantly associated with mortality. Our results suggest that severe forms of mSSCI should be avoided and normal levels of blood glucose, sodium, and carbon dioxide should be targeted in neonates with HIE. Close monitoring and regular adjustments are therefore necessary.

## Data Availability

The datasets presented in this article are not readily available because data can be shared on request and upon authorization of the institution. Requests to access the datasets should be directed to isabelle.guellec@univ-cotedazur.fr.

## References

[1] Mathew JL Kaur N Dsouza JM. Therapeutic hypothermia in neonatal hypoxic encephalopathy: a systematic review and meta-analysis. J Glob Health. (2022) 12:04030. 10.7189/jogh.12.04030 35444799 PMC8994481

[2] Geeraerts T Velly L Abdennour L Asehnoune K Audibert G Bouzat P Management of severe traumatic brain injury (first 24 h). Anaesth Crit Care Pain Med. (2018) 37(2):171–86. 10.1016/j.accpm.2017.12.001 29288841

[3] Collège de la Haute Autorité de Santé. avc_prise_en_charge_precoce_-_argumentaire.pdf Available online at: https://www.has-sante.fr/upload/docs/application/pdf/2009-07/avc_prise_en_charge_precoce_-_argumentaire.pdf (Accessed February 5, 2021).

[4] Sharma D Smith M. The intensive care management of acute ischaemic stroke. Curr Opin Crit Care. (2022) 28(2):157. 10.1097/MCC.0000000000000912 35034076

[5] Guellec I Ancel PY Beck J Loron G Chevallier M Pierrat V Glycemia and neonatal encephalopathy: outcomes in the LyTONEPAL (long-term outcome of neonatal hypoxic EncePhALopathy in the era of neuroprotective treatment with hypothermia) cohort. J Pediatr. (2023) 257:113350. 10.1016/j.jpeds.2023.02.003 36828343

[6] Montaldo P Caredda E Pugliese U Zanfardino A Delehaye C Inserra E Continuous glucose monitoring profile during therapeutic hypothermia in encephalopathic infants with unfavorable outcome. Pediatr Res. (2020) 88(2):218–24. 10.1038/s41390-020-0827-4 32120381

[7] Puzone S Diplomatico M Caredda E Maietta A Giudice EMD Montaldo P. Hypoglycaemia and hyperglycaemia in neonatal encephalopathy: a systematic review and meta-analysis. Arch Dis Child Fetal Neonatal Ed. (2023) 109:18–25. 10.1136/archdischild-2023-325592 37316160

[8] Nadeem M Murray D Boylan G Dempsey EM Ryan CA. Blood carbon dioxide levels and adverse outcome in neonatal hypoxic-ischemic encephalopathy. Am J Perinatol. (2010) 27(5):361–5. 10.1055/s-0029-1243309 20013576

[9] Pappas A Shankaran S Laptook AR Langer JC Bara R Ehrenkranz RA Hypocarbia and adverse outcome in neonatal hypoxic-ischemic encephalopathy. J Pediatr. (2011) 158(5):752–758.e1. 10.1016/j.jpeds.2010.10.019 21146184 PMC3229432

[10] Lingappan K Kaiser JR Srinivasan C Gunn AJ. Relationship between PCO2 and unfavorable outcome in infants with moderate-to-severe hypoxic ischemic encephalopathy. Pediatr Res. (2016) 80(2):204–8. 10.1038/pr.2016.62 27049290

[11] Thakur J Bhatta NK Singh RR Poudel P Lamsal M Shakya A. Prevalence of electrolyte disturbances in perinatal asphyxia: a prospective study. Ital J Pediatr. (2018) 44(1):56. 10.1186/s13052-018-0496-7 29784025 PMC5963047

[12] Devi U Pullattayil AK Chandrasekaran M. Hypocarbia is associated with adverse outcomes in hypoxic ischaemic encephalopathy (HIE). Acta Paediatr. (2023) 112(4):635–41. 10.1111/apa.16679 36662594

[13] Ottolini KM Basu SK Herrera N Govindan V Mashat S Vezina G Positive fluid balance is associated with death and severity of brain injury in neonates with hypoxic-ischemic encephalopathy. J Perinatol Off J Calif Perinat Assoc. (2021) 41:1331–8. 10.1038/s41372-021-00988-w PMC10363283 33649446

[14] Meau-Petit V Tasseau A Lebail F Ayachi A Layouni I Patkai J Hypothermie contrôlée du nouveau-né à terme après asphyxie périnatale. Arch Pédiatrie. (2010) 17(3):282–9. 10.1016/j.arcped.2009.10.030 20144852

[15] Sarnat HB Sarnat MS. Neonatal encephalopathy following fetal distress. A clinical and electroencephalographic study. Arch Neurol. (1976) 33(10):696–705. 10.1001/archneur.1976.00500100030012 987769

[16] Xu EH Claveau M Yoon EW Barrington KJ Mohammad K Shah PS Neonates with hypoxic-ischemic encephalopathy treated with hypothermia: observations in a large Canadian population and determinants of death and/or brain injury. J Neonatal-Perinat Med. (2020) 13(4):449–58. 10.3233/NPM-190368 32310192

[17] Harding JE Harris DL Hegarty JE Alsweiler JM McKinlay CJ. An emerging evidence base for the management of neonatal hypoglycaemia. Early Hum Dev. (2017) 104:51–6. 10.1016/j.earlhumdev.2016.12.009 27989586 PMC5280577

[18] Basu SK Ottolini K Govindan V Mashat S Vezina G Wang Y Early glycemic profile is associated with brain injury patterns on magnetic resonance imaging in hypoxic ischemic encephalopathy. J Pediatr. (2018) 203:137–43. 10.1016/j.jpeds.2018.07.041 30197201 PMC6323004

[19] Édition professionnelle du Manuel MSD. Hyponatrémie néonatale - Pédiatrie. Available online at: https://www.msdmanuals.com/fr/professional/p%C3%A9diatrie/troubles-m%C3%A9taboliques-%C3%A9lectrolytiques-et-toxiques-chez-le-nouveau-n%C3%A9/hyponatr%C3%A9mie-n%C3%A9onatale?query=hyponatr%C3%A9mie%20n%C3%A9onatale (Accessed March 7, 2021).

[20] Wong SK Chim M Allen J Butler A Tyrrell J Hurley T Carbon dioxide levels in neonates: what are safe parameters? Pediatr Res. (2022) 91(5):1049–56. 10.1038/s41390-021-01473-y 34230621 PMC9122818

[21] Nadeem M Murray DM Boylan GB Dempsey EM Ryan CA. Early blood glucose profile and neurodevelopmental outcome at two years in neonatal hypoxic-ischaemic encephalopathy. BMC Pediatr. (2011) 11:10. 10.1186/1471-2431-11-10 21294901 PMC3040139

[22] Paulsen ME Rao RB. Cerebral effects of neonatal dysglycemia. Clin Perinatol. (2022) 49(2):405–26. 10.1016/j.clp.2022.02.008 35659094 PMC9177056

[23] Stanley CA Thornton PS Leon D D D. New approaches to screening and management of neonatal hypoglycemia based on improved understanding of the molecular mechanism of hypoglycemia. Front Pediatr. (2023) 11:1071206. 10.3389/fped.2023.1071206 36969273 PMC10036912

[24] Szakmar E Munster C El-Shibiny H Jermendy A Inder T El-Dib M. Hypocapnia in early hours of life is associated with brain injury in moderate to severe neonatal encephalopathy. J Perinatol Off J Calif Perinat Assoc. (2022) 42(7):892–7. 10.1038/s41372-022-01398-2 35461333

[25] Lopez Laporte MA Wang H Sanon PN Barbosa Vargas S Maluorni J Rampakakis E Association between hypocapnia and ventilation during the first days of life and brain injury in asphyxiated newborns treated with hypothermia. J Matern Fetal Neonatal Med. (2019) 32(8):1312–20. 10.1080/14767058.2017.1404980 29129133

[26] Basu P Som S Das H Choudhuri N. Electrolyte status in birth asphyxia. Indian J Pediatr. (2010) 77(3):259–62. 10.1007/s12098-010-0034-0 20177828

[27] Gupta BD Sharma P Bagla J Parakh M Soni JP. Renal failure in asphyxiated neonates. Indian Pediatr. (2005) 42(9):928–34. PMID: 16208054. 16208054

[28] Wintermark P Mohammad K Bonifacio SL. Proposing a care practice bundle for neonatal encephalopathy during therapeutic hypothermia. Semin Fetal Neonatal Med. (2021) 26(5):101303. 10.1016/j.siny.2021.101303 34711527

